# Tooth retention predicts good physical performance in older adults

**DOI:** 10.1371/journal.pone.0255741

**Published:** 2021-09-20

**Authors:** Estella Musacchio, Pierluigi Binotto, Egle Perissinotto, Giuseppe Sergi, Sabina Zambon, Maria-Chiara Corti, Anna-Chiara Frigo, Leonardo Sartori

**Affiliations:** 1 Department of Medicine DIMED, University of Padova, Padova, Italy; 2 Dental Private Practice, Padova, Italy; 3 Department of Cardiologic, Thoracic and Vascular Sciences - Unit of Biostatistics, Epidemiology and Public Health - University of Padova, Padova, Italy; 4 Azienda Zero, Epidemiology Division, Padova, Italy; Cardiff University, UNITED KINGDOM

## Abstract

**Background:**

Oral health is closely related to both physical and psychological well-being, as it enables individuals to eat, speak, and socialize. The number of teeth is the most used indicator of oral health. Several reports document a relationship of dental status with a variety of indicators of general health but longitudinal studies employing standardized physical performance tests are infrequent in the scientific literature.

**Subjects and methods:**

The Italian elderly participating in the Pro.V.A. longitudinal Study (3099 subjects aged 65+ at baseline, 2196 at the 5-year follow-up 1 and 1641 at the 7-year follow- up 2) underwent detailed interview and extensive clinical and instrumental examination that included validated physical performance measures. Participants were classified into 4 groups according to the number of remaining teeth: 0, 1–7, 8–19, and 20+. To explore the association of the number of remaining teeth with physical function and disability, we performed logistic regression analyses with models progressively adjusted for a wide number of covariates, namely anthropometric (gender, age, BMI), comorbidity (cardio-vascular, osteoarticular, and neurological diseases including depression), muscle strength (assessed for upper and lower limbs), lifestyle (smoking status, alcohol use, leisure time activities) and socioeconomical status (education, income, marital status, loneliness).

**Results:**

Dental status correlated with most comorbidities, lifestyle, and socio-economic variables at the univariate analysis at baseline and at follow-ups. A good dental status was significantly associated with better physical functioning and lower disability. The presence of 20+ teeth resulted significantly protective (reference group: 0 teeth) versus mobility-related disability (OR = 0.67), disability (OR = 0.54) and inability to perform heavy duties (OR = 0.62), at follow up 1 and low physical performance score (OR = 0.59) at follow up 2. Conversely, the detrimental effect of edentulism, explored in subjects with or without dentures, was present but not as straightforward. Conclusion. The assessment of a geriatric patient should include an oral evaluation as a good dental status is a crucial component of successful aging.

## Introduction

Back in 1987, Rowe and Kahn highlighted that the distinction between presence or absence of diseases and disabilities may be insufficient to describe the actual condition of an aging subject. Consequently, they proposed a multidimensional approach of the nonpathological elder that included physical and cognitive functions evaluation thus allowing a more appropriate distinction of aging in “usual”, i.e. non pathologic but high risk, and” successful”, i.e. low risk and high function [1,2]. The concept of successful aging has been long debated after since, mostly focusing on how to expand functional years in a later life span [3]. Oral health is closely related to both physical and psychological well-being, as it enables individuals to eat, speak, and socialize [4]. The most used indicator of oral health is the number of teeth. Tooth loss has been demonstrated to be associated with several comorbidities, in particular cardiovascular events (CVD)—including coronary heart disease, stroke/TIA, hypertension [5]—cognitive decline [6], and respiratory diseases [7]. A poor oral status could impair taste and the ability to chew thus limiting food choices and compromising the digestive process. Moreover, it affects the subject’s aesthetics and phonetics often resulting in limited social and personal interaction. Therefore full or partial edentulism represents a risk factor for malnutrition, quality of life deterioration, loss of self-sufficiency, and disability **[**8,9]. A recent systematic review identified significant longitudinal associations between oral health indicators and frailty, an important geriatric health outcome that includes physical components, such as handgrip strength, walking speed and physical activity [10]. On the other hand, several reports document a relationship of a good dentition with a variety of indicators of both health and longevity [11], but longitudinal studies employing validated physical performance tests are infrequent in the scientific literature. The aim of the present study was to investigate the association of the number of remaining teeth with physical functioning and disability in aging Italian men and women evaluated in the Progetto Veneto Anziani (Pro.V.A.) longitudinal Study, by means of standardized physical performance measures, with progressive adjustments with many covariates in order to exclude possible intermediates in the relationship.

## Materials and methods

### Data source and subjects

The Pro.V.A. Study is an observational cohort study of an Italian population aged ≥65 living in Northeastern Italy, designed to focus on the effects of cardiovascular and osteoarticular disease on disability. The design and the sampling frame of the study have been previously described in detail elsewhere [12]. Briefly, the study population included 3099 age- and sex-stratified white participants (1245 men and 1854 women, age range 65–101), who were randomly selected between 1995 and 1997 using a multistage stratified method. The baseline assessment was followed by two in-person follow-up (FU) examinations (5 and 7 years later) with ongoing mortality and morbidity surveillance. The Pro.V.A. Study design and protocol were approved by the two relevant Institutional Review Boards of the Veneto Region Health Care Agencies. All the participants signed an informed written consent form, were interviewed at their homes, and examined by specially trained physicians and nurses who followed a protocol consisting of an extensive battery of clinical, instrumental, biochemical and physical performance tests, at the two clinic facilities provided for the study’s implementation.

Of the 3099 older adults initially enrolled in the study, 3083 had data concerning their oral, comorbidity and disability status, and leisure time activities. Complete baseline physical performance information was available for 2083 participants. The subjects lost to FU1 included 739 people who died, 312 who missed one or more physical functioning data, and 148 dropouts. The latter were comparable with the subjects who remained in the study, while the other participants lost to FU1 had lower number of teeth, worse physical performance, and a higher prevalence of disability, comorbidities and sedentarism. The proportion of women was highest in the group with missing FU measures (72.8%) and the lowest among the deceased (48.9%). Attrition data between FU2 and FU1 overlapped with those between baseline and FU1. Specifically, 708 had died and there were 301 between the dropouts and the subjects without complete information: all were older, had fewer teeth and more disabilities. Women were more frequent among the participants with missing data.

### Dentition assessment

During the physical work-up at the Study centers, trained clinicians State-board qualified to practice dentistry performed an oral examination to assess each patient’s general oral status and number of teeth. All teeth regardless of their condition (sound, decayed, or treated with any kind of restoration) were counted when the total number of teeth was being calculated. The presence of an upper and/or lower removable prosthesis was also recorded. Information on self-reported difficulty in chewing and in swallowing was obtained during the home interview by means of two specific questions. The subjects were divided into one of 4 classes depending on their dentition at baseline: edentulous, 1–7 teeth, 8–19 teeth, and 20+ teeth [13].

### Assessment and classification of disability and physical functioning

Information on the subject’s history of falls, difficulty in activities of daily living (ADL), and physical activity habits was collected during an in-person or proxy interview. Physical performance measures were carried out during the home interview, muscle strength tests were carried out at the Study centers. Disability in ADL was defined as the need for help or the inability to perform one or more of the following activities: walking across a room (ADL1), bathing (ADL2), dressing (ADL3), eating (ADL4), transferring from bed to chair (ADL5) or using the toilet (ADL6).

The subjects’ physical activity level was figured on the basis of their response to three questions regarding frequency of walking 500 m, riding a bike > 1 km, and gardening. Those who reported never doing these three activities were classified as “sedentary”, those who reported engaging in at least two of these activities every day or more than once a day as “active”, and those with an activity level somewhere in between as “intermediate” [14]. Lower extremity function was evaluated by means of tests assessing: standing balance (side-by-side, semitandem, and tandem tests), normal walking speed (3 m), and the ability to repeatedly (5 times) rise from a chair, using the short physical performance battery (SPPB) developed and validated by Guralnik [15]. A score of 0 was assigned to those who did not complete the test, while scores from 1 to 4 were given to each category of test, assigning 4 to the best performance. The three tests of standing balance were considered as a single test, with hierarchical difficulty (those who could hold the side-by-side position passed on to the semitandem and eventually to the tandem, each position had to be hold for 10 sec). For walking speed and chair stands, time of performance was categorized in quartiles, assigning the highest score to those who were within the lowest time quartile. Baselines values (in sec) for the first quartile were 4.6 and 4.3 for the two walking tests, 3.1 for rapid march, and 9.8 for chair raising; at FU1 they were: 4.5 and 4.3, 3.1 and 10.8 respectively; at FU2: 4.3 and 4.2, 3.0 and 11.2. Participants were classified using a summary performance score by adding the categorical scores described. For the analyses, SPPB score was dichotomized at 9, corresponding to the 3^rd^ quartile of the studied population at all time points of the study.

Overall physical performance was classified in accordance with Guralnik’s model [16,17] using a three-level hierarchical scale regarding mobility-related disability (MRD) containing a subset of questions from a scale originally developed by Berkman [18]: no disability; MRD only, i.e. inability to walk 0.5 mile or to climb stairs without help; MRD plus disability in performing at least one ADL.

Summarizing, the physical and disability outcome variables at baseline, FU1 and FU2 were dichotomized as follows: i) limited lower extremity function, assessed by mean of the short physical performance battery (SPPB): score ≥9; ii) mobility-related disability: three categories at baseline (0 = no MRD; 1 = MRD only; 2 = MRD + disability in ≥1 ADL) was dichotomized at FUs as no subjects were in category 0; iii) physical activity level (sedentarism): none vs moderate/intense; iv) disability in activities of daily living (ADL): 0–1 vs >1; v) (in)ability to perform heavy duties only assessed at baseline FU1.

### Anthropometric measures, nutritional status and disease adjudication

Body mass index (BMI), a general nutrition index, and serum albumin, a biomarker specific for protein energy malnutrition, were used to describe nutritional status. The former is a widely applied index in geriatric clinical practice, mainly used to identify subjects at risk for under-nutrition. The latter is considered an index of long-term malnutrition as its half-life is the longest among all circulating proteins [19]. Serum albumin levels were assessed in fasting blood samples. In view of the fact that low albumin levels are uncommon in elderly Italian populations, hypoalbuminemia was defined as values lower than 4.0 g/dL [8]. Vitamin D levels were categorized in sex-specific quartiles of 25-OH calciferol serum levels, considering hypovitaminosis values below the first quartile. Hypertension was defined as systolic blood pressure ≥140 mmHg and/or diastolic blood pressure ≥90 mm Hg; blood pressure <130 and <80 mmHg was considered normal. Hypotension (systolic pressure below 90 and diastolic below 60 mmHg) was neglectable (<1.5%) in the study population. Diseases present at baseline and/or at the FU assessments were identified by the physicians on the basis of the data recorded during the initial interview, the physical examination, hospital records, medical surveillance, use of medications, X-ray readings, and results of blood assays collected on the participant. Disease adjudication was made for: angina, myocardial infarction, congestive heart failure, peripheral artheriopathy, stroke, femoral fracture, osteoporosis, osteoarthritis (separately for hands, knees, hips), rheumatoid arthritis, Parkinson’s disease, bronchopneumopathy, diabetes, neoplasm, visual impairment, hearing impairment.

Depression was evaluated using the 30-item depression geriatric scale (GDS) [20]; a score higher than 15 denoted severe depression. Cognitive status was assessed during the home interview by administering the 30-items questionnaire of the Mini Mental State Examination, with cognitive impairment corresponding to a score <18 [21] In the latter case the test was repeated for confirmation by the physician at the Study center during the medical evaluation.

### Socio economic status and lifestyle factors

The information regarding socio-economic status (SES) and lifestyle factors, which was self-reported, was gathered during the baseline interview. The following surrogate variables were used for the subject’s socio-economic condition: monthly income (<500 €, ≥ 500 €), living alone (yes/no), widowhood (yes/no), educational level (categorized at 5 years of school attended, compulsory education for elderly subjects), number of rooms in the house (dichotomized at 5). The final SES variable was dichotomized, assigning 0 for 0–1 adverse conditions. Smoking status was assessed during the home interview using seven specific questions concerning current or past smoking patterns, type of smoking, the mean number of cigarettes or other per day, age of the participant at beginning, age at quitting (when appropriate). Smoking status was categorized here as dichotomous: yes (current smokers) or no (either never or former smokers). Alcohol consumption was defined as one bottle/can of beer, one glass of wine, one cocktail, one shot of liquor. Drinking was considered a dichotomous variable and a non-drinker someone who did not consume alcohol over the last 30 days. Information on SES and lifestyle factors was obtained from 99% of the participants.

### Statistical analysis

The normality of the continuous variables was verified using the Shapiro-Walk test. Quantitative variables were summarized as mean ± standard deviation or median with minimum and maximum; qualitative ones as frequency distributions. Analysis of variance or *t* test was used to compare mean values among groups for normally distributed variables and non parametric Mann-Whitney test for non normal variables. The Chi square test was used to compare categorical distributions.

At FU1 and FU2, mortality was computed according to teeth class. Separate logistic regression models were performed to estimate the independent contribution (adjusted ORs and the respective 95% CI) of the baseline dental status to the different outcome (SPPB score<9, MRD, Sedentarism, Disability in ADL; Inability to perform heavy duties) progressively adjusting (from Model 1 to model 7) for conditions at baseline. In the models, dental status was entered as categorized in four classes (edentulous, 1–7 teeth, 8–19 teeth, 20+ teeth), reference group was 0 teeth. All the other variables were entered as dichotomous, except for age that was categorical (three levels: 65–74, 75–84, 85+ yrs) and BMI that was continuous. In the progressively adjusted models, besides the baseline tooth class, the following covariates were entered: Model 1: sex, age, and BMI; Model 2: Model 1 + comorbidity (more than one of: myocardial infarction, angina, stroke, congestive heart failure, peripheral arteriopathy, hypertension, chronic obstructive bronchopneumopathy, diabetes, neoplasm) + osteoarthritis, osteoporosis, femoral fracture, history of falling, visual impairment, hearing impairment; Model 3: Model 2 + cognitive impairment, depression, smoking status, alcohol intake, and social variables; Model 4: Model 3 + serum albumin level, serum vitamin D level, neurological impairment (including Parkinson disease), hand strength, leg strength; Model 5: Model 4 + basal physical activity; Model 6: Model 5 + difficulty at chewing and at swallowing; Model 7: Model 6 + value of the model outcome at baseline (for FU1) or at FU1 (for FU2). For the study of the impact of edentulism without dentures, the variable was dichotomized (edentulous with no dentures vs all the others). Degree of collinearity among covariates was estimated using the variance inflation factor (VIF, exclusion cut off = 2). Only the data on subjects with complete dentition records were included in our analyses. A *p* level lower than 0.05 was considered significant. All statistical analyses were performed using the R Statistical Software [22].

## Results

Data about oral conditions were obtained from 3083 subjects (1237 M, 1846 F) at baseline, from 2196 survivors (805 M, 1391 F) at the first FU examination, and from 1640 (576 M, 1064 F) at the second one.

The main features of the study population at the three time points of the study, according to tooth classes at baseline, are shown in Table 1. Most variables were significantly different among groups, with subjects with fewer teeth being the older.

**Table 1:** Population characteristics at baseline, follow up 1, and follow up 2 according to baseline tooth class.

	Overall	0 teeth	1–7 teeth	8–19 teeth	20+ teeth	p
**Baseline**						
Demo-anthropometric						
n	3052	1333	586	651	482	
Males (%)	40.1	37.9	39.4	41.8	45.0	<0.001
Age (yrs)	75 (65–101)	78 (65–99)	76 (65–101)	74 (65–96)	71 (65–91)	<0.001
BMI (kg/m^2^)	27.2 (14.7–54.3)	27.0 (14.7–46.5)	26.8 (15.1–45.0)	27.78 (15.7–54.3)	27.4 (17.6–53.6)	<0.001
Comorbidities (%)						
Angina	4.3	5.0	3.1	3.2	5.1	0.076
Myocardial Infarction	4.6	5.1	4.4	3.8	4.5	0.617
Stroke	4.8	6.8	3.7	2.9	3.3	<0.001
Peripheral arteriopathy	15.3	19.9	15.4	11.3	7.4	<0.001
Congestive heart failure	7.0	8.9	8.3	4.6	3.3	<0.001
Osteoporosis	41.5	44.8	42.1	39.8	34.3	0.001
Osteoarthritis	25.7	29.8	24.9	23.0	19.3	<0.001
Neoplasm	7.7	7.4	8.1	7.3	8.8	0.726
Diabetes	10.0	11.0	9.3	9.9	8.2	0.332
Chronic obstructive bronchopneumopathy	9.6	11.6	9.3	7.5	7.0	0.003
Hearing impairment	56.4	61.7	57.2	54.2	44.0	<0.001
Visual impairment	25.3	29.5	25.8	22.5	16.5	<0.001
Parkinson’s disease	1.3	1.5	0.8	1.1	1.2	0.668
Cognitive impairment	10.4	14.2	12.5	5.9	2.7	<0.001
Depression	16.5	17.6	16.9	15.2	15.0	0.461
History of falling	30.8	31.4	35.9	28.8	25.5	0.002
Femoral fracture	3.8	4.3	3.2	3.1	4.1	0.485
Vitamin D <1^st^ quart	8.3	9.5	11.1	6.4	3.8	<0.001
Serum albumin (g/dL)	4.3±0.4	4.3±0.4	4.3±0.4	4.4±0.4	4.4±0.4	<0.001
Lifestyle and socioeconomic (%)						
Alcohol use	79.1	76.7	77.9	80.2	85.9	<0.001
Current smoking	37.8	39.6	36.2	36.6	36.7	0.386
Low income	61.0	61.2	66.1	59.6	56.1	0.008
Low education	94.4	96.1	96.1	93.3	89.3	<0.001
Living alone	17.2	18.9	14.8	17.6	14.7	0.068
Widowhood	41.1	46.4	42.1	39.6	27.2	<0.001
**Follow up 1**						
Demo-anthropometric						
n	2176	1006	413	473	284	
Males (%)	36.8	38.0	39.5	41.4	45.2	0.039
Age (yrs)	78 (69–102)	80 (69–102)	79 (69–100)	78 (69–97)	75 (69–94)	<0.001
BMI (kg/m^2^)	27.2 (14.5–68.7)	27.0 (14.5–65.2)	26.9 (15.2–57.4)	27.7 (15.4–68.7)	27.6 (17.6–58.7)	0.002
Comorbidities (%)						
Angina	3.8	3.3	4.0	3.3	5.3	0.354
Myocardial Infarction	4.1	3.4	5.3	4.0	4.5	0.457
Stroke	3.3	3.9	3.8	2.5	2.8	0.486
Peripheral arteriopathy	16.4	20.0	16.5	14.1	10.8	<0.001
Congestive heart failure	13.2	16.8	12.8	11.2	8.0	<0.001
Osteoporosis	21.7	23.3	21.1	20.6	20.1	0.479
Osteoarthritis	28.0	32.2	27.6	26.4	21.1	<0.001
Neoplasm	7.6	7.8	9.3	6.7	6.8	0.459
Diabetes	12.7	12.5	13.8	12.7	12.0	0.892
Chronic obstructive bronchopneumopathy	9.9	12.8	9.3	7.1	7.3	0.001
Hearing impairment	na					
Visual impairment	22.7	26.6	23.3	19.3	17.0	<0.001
Parkinson’s disease	1.8	2.5	1.5	1.2	1.0	0.149
Cognitive impair	14.0	18.8	16.6	10.2	5.0	<0.001
Depression	22.8	23.0	24.9	22.2	20.9	0.501
History of falling	36.8	38.5	41.0	33.8	32.2	0.038
Femoral fracture	2.7	3.4	3.0	2.1	1.3	0.115
Vitamin D <1^st^ quart	na					
Serum albumin (g/dL)	4.0±0.4	4.0±0.4	4.0±0.4	4.0±0.4	4.1±0.4	<0.001
Lifestyle and socioeconomic (%)						
Widowhood	47.1	53.1	48.4	46.7	31.9	<0.001
Institutionalized	2.7	3.3	3.4	1.9	1.5	0.129
Dead	23.8	27.9	26.5	20.7	13.3	<0.001
**Follow up 2**						
Demo-anthropometric						
n	1641	737	333	356	215	
Males (%)	35.2	38.0	39.5	41.4	45.2	0.040
Age (yrs)	79 (71–102)	81 (71–102)	80 (71–100)	78 (71–98)	77 (71–96)	<0.001
BMI (kg/m^2^)	27.5 (16.2–57.1)	27.4 (16.2–44.3)	27.7 (17.3–44.3)	27.8 (17.2–53.9)	27.5 (16.9–57.1)	0.800
Comorbidities (%)						
Angina	3.0	3.2	2.2	1.1	5.4	0.007
Myocardial Infarction	2.2	2.4	2.5	2.9	0.9	0.287
Stroke	1.9	1.9	1.9	0.8	3.0	0.202
Peripheral arteriopathy	17.6	19.9	21.8	16.2	10.7	0.001
Congestive heart failure	11.6	14.6	8.5	11.4	9.0	0.013
Osteoporosis	22.1	23.0	20.9	22.1	21.5	0.890
Osteoarthritis	19.6	24.1	24.4	16.4	10.7	<0.001
Neoplasm	4.4	5.9	3.5	3.5	3.3	0.111
Diabetes	13.3	14.1	14.6	13.3	10.7	0.439
Chronic obstructive bronchopneumopathy	10.4	12.5	12.7	8.2	6.9	0.011
Hearing impairment	na					
Visual impairment	20.6	22.5	25.3	19.7	13.7	0.001
Parkinson’s disease	2.1	3.1	2.2	1.1	1.2	0.103
Cognitive impairment	12.8	17.8	17.7	8.5	3.6	<0.001
Depression	8.8	8.4	10.8	8.5	8.1	0.587
History of falling	33.6	37.5	34.2	31.9	28.0	0.024
Femoral fracture	1.7	2.6	1.9	1.1	0.6	0.099
Vitamin D <1^st^ quart	na					
Serum albumin (g/dL)	4.1±0.4	4.1±0.4	4.1±0.4	4.1±0.4	4.2±0.3	0.002
Lifestyle and socioeconomic (%)						
Widowhood	na					
Institutionalized	0.8	0.5	1.0	1.1	0.9	0.777
Dead	30.0	36.1	27.1	28.1	20.3	<0.001

na = not assessed.

Tooth retention, use of removable prosthesis, and the prevalence of self-reported difficulty at chewing or swallowing in the two genders at baseline and FUs is outlined in detail in Table 2. The prevalence of edentulism in the total population reached 48.2% at the time of FU2 assessment and was more pronounced in women at all the time points (p<0.05 at baseline and FU1, p<0.005 at FU2). Incident edentulism was 14.7% and 21.8% at FU1 and FU2 respectively vs baseline, similar in the two genders. The average number of lost teeth was instead different, significantly higher in men. The use of both upper and lower prostheses was quite frequent, but the prevalence of edentulous subjects not wearing dentures, more common in women (p<0.005 at FU1), was around 8%. It decreased at FU1, possibly as a consequence of older age and higher mortality in this category. Adjustment for age, performed to better interpret this finding, disclosed an increased mortality risk for edentulous not wearing dentures at both FUs, with ORs (95% CI): 1.88 (1.39–2.53), p<0.0001 and 1.91 (1.28–2.85), p<0.005 at FU1 and FU2 respectively (data not shown). Self-reported difficulty at swallowing and at chewing were significantly more prevalent in women at all time points, the latter in particular (p<0.0001). Table 3 describes the behavior of physical performance and disability variables according to tooth classes, at baseline and follow ups. The prevalence of disability was lower and physical performance progressively better in subjects who retained teeth both at baseline and at the two FU assessments. Participants reporting mobility-related disability plus disability in at least one ADL in the population as a whole were 31.9% (n = 989) at baseline, 59.6% at the first FU, and 64.6% at the second one. The difference between subjects with 0 and 20+ teeth was quite remarkable (38.3% vs 17.7%, 68.8% vs 41.5% and 72.6% vs 46.7% respectively). The percent of participants reporting no MRD disability was 43.6% (n = 1351) at baseline, but there were no participants in this category at the subsequent FUs. At both follow ups, mean SPPB scores were significantly lower in edentulous subjects not wearing dentures (5.9±3.6 and 6.0±3.5 at FU1 and FU2 respectively) compared to those who did (7.1±3.1 and 6.9±3.1) and progressively higher in subjects retaining teeth (7.2±3.2 and 7.0±3.3 at FU1 and FU2 for those retaining 1–7 teeth vs 8.4±2.7 and 8.3±3.0 respectively for those with 20+ teeth, p<0.001 across classes). As expected, mean age significantly increased by decreasing tooth number. Table 4 presents the ORs and 95% CIs of the logistic regression analyses using progressively adjusted models to determine the independent contribution of dental status to physical activity and performance at the FU assessments. The table reports only ORs specific for the 20+ teeth class, as in this group the associations were clearer and more robust. Up to Model 4 the adjustments considered anthropometric and sociodemographic variables, lifestyle (including smoking status and alcohol use), comorbidities and muscle strength, as detailed in the methods. Model 5 was adjusted for the frequency of physical activity at baseline, in order to exclude biases due to the subject’s attitude rather than capability. A 20+ teeth dentition was positively associated with all the considered physical function outcomes, considered at FU1 and 2, with 0 teeth being the reference group. Up to Model 6, adjusted also for difficulty at chewing and at swallowing, it resulted protective versus a low SPPB score (OR = 0.60 and 0.55 at FU1 and 2, respectively, p<0.005), MRD (OR = 0.62 and 0.65 at FU1 and 2, respectively, p<0.02), sedentarism at FU2 (OR = 0.58, p<0.03), and inability to perform heavy duties determined only at FU1 (p<005), as unfortunately the variable was not collected at FU2. A final adjustment was done to verify the contribution of a good dental status to the preservation of a satisfactory physical function. To this aim, for each outcome, the model was further adjusted for the corresponding variable at the previous time point (baseline for FU1 outcomes, and FU1 for FU2). A dentition comprising 20+ teeth resulted protective for the decline of SPPB score at FU2 (OR = 0.59, p<0.02), and MRD, disability, and the ability to perform heavy duties at FU1 (OR = 0.67, p<0.05; OR = 0.54 p = 0.007; OR = 0.62, p = 0.006 respectively). In most of the cases of non statistically significant ORs, 95% CI was just above 1.00, indicating that the relationship was present, though marginally. Interestingly, for SPPB score at FU2 and MRD at FU1, the 8–19 teeth class was also significantly associated throughout all models. On the contrary, the association of edentulism with physical functioning was present and remarkable but not as consistent throughout the adjusted models as that of good dentition (data not shown). In order to evaluate the possible role of total or partial edentulism as a risk factor for poor physical performance and disability, other analyses were done taking also into account the use of prostheses. The condition of edentulism with no dentures, though resulting as a risk factor for mobility related disability at FU1 in the fully adjusted Model 7 (OR:95% CI, 2.58:1.04–6.40), did not show strong associations with the other outcomes.

**Table 2:** Gender differences in dentition-related characteristics at baseline and follow ups.

	Females			Males			Total population		
	B (n = 1846)	FU1 (n = 1391)	FU2 (n = 1064)	B (n = 1237)	FU1 (n = 805)	FU2 (n = 576)	B (n = 3083)	FU1 (n = 2196)	FU2 (n = 1640)
teeth n (%)									
0 teeth	837 (45.3)	703 (50.5)	539 (50.7)	512 (41.4)*	360 (44.7)*	251 (43.6)**	1349 (43.8)	1063 (48.4)	790 (48.2)
1–7 teeth	357 (19.3)	251 (18.0)	208 (19.5)	233 (18.8)	143 (17.8)	104 (18.1)	590 (19.1)	394 (17.9)	312 (17.9)
8–19 teeth	385 (20.9)	279 (20.1)	204 (19.2)	272 (22.0)	188 (23.4)	137 (23.8)	657 (21.3)	467 (21.3)	341 (20.8)
20+ teeth	267 (14.5)	158 (11.4)	113 (10.6)	220 (17.8)	114 (14.2)	84 (14.6)	487 (15.8)	272 (12.4)	197 (12.0)
prosthesis use (%)									
lower only	3.0	4.2	3.9	3.6	5.1	4.5	3.2	4.6	4.1
upper only	8.8	8.9	8.8	6.1	7.7	8.2	7.7	8.6	8.6
both	50.0	53.0	57.5	48.2	51.8	52.5	49.3	53.6	55.8
none	38.3	33.9	29.8	42.1	35.4	34.8	39.8	35.1	31.6
0 teeth no prosthesis	8.0	9.3	7.0	6.9	6.1*	4.9	7.6	8.2	6.3
lost teeth (n mean±SD) [n median (min-max)]									
difference B-FU1	1.42±2.84 [0 (0–22)]			1.84±3.35 [0 (0–27)^§^]				1.58±3.04	
difference B-FU2	2.16±3.76 [0 (0–24)]			2.79±4.26 [1 (0–26) ^§§^]				2.38±3.95	
difference FU1-FU2	0.74±2.24 [0 (0–24)]			0.94±2.34 [0 (0–26) ^§^]				0.81±2.28	
difficulty (%)									
at chewing	51.7	50.9	50.4	41.3^§§^	39.9^§§^	38.9^§§^	47.5	46.9	46.4
at swallowing	15.5	17.8	17.8	11.0^§§^	14.1*	12.0**	13.7	16.4	15.8

B = baseline; FU1 = follow up 1; FU2 = follow up 2; p (M vs F):

*<0.05;

**<0.005;

^§^ 0.001;

^§§^<0.001.

**Table 3:** Items of physical performance and disability according to teeth classes at baseline and follow ups.

**a. Baseline**		teeth class				
		0 1349	1–7 590	8–19 657	20+ 487	p
Physical activity (%)	intense sedentary	16.5 31.7	19.7 28.0	22.8 18.9	23.2 14.8	<0.001
Disability in ADL (%)	0–1 >1 6	69.3 30.7 7.5	72.5 27.5 5.6	80.1 19.9 3.7	86.2 13.8 1.4	<0.001
Mobility-related disability (%)	No MRD MRD alone MRD +ADL	35.9 25.9 38.3	42.0 22.0 35.9	51.2 23.0 25.8	57.0 25.3 17.7	<0.001
Normal speed march 3m (sec)	mean ±SD median (min-max)	6.41±3.8 5.4 (2.7–59.8)	6.58±4.6 5.5 (2.7–60.2)	5.96±3.7 5.1 (2.3–48.0)	5.36±2.9 4.8 (2.0–31.8)	<0.001
Rapid march 3m (sec)	mean ±SD median (min-max)	4.58±3.0 3.9 (2.0–63.1)	4.45±2.1 3.9 (1.9–25.5)	4.16±2.5 3.6 (1.0–31.0)	3.78±1.7 3.5 (2.0–27.7)	<0.001
Chair stands 5 times (sec)	mean ±SD median (min-max)	13.9±7.8 12.4 (3.0–151.0)	13.6±8.3 11.95 (5.3–129.0)	12.8±6.7 11.50 (5.5–115.0)	12.1±5.4 11.0 (5.1–92.8)	<0.001
Standing balance test*	mean ±SD % scoring 4	3.39±0.8 55.1	3.41±0.9 59.9	3.57±0.8 68.7	3.7±0.7 78.8	<0.001
Summary SPPB score**	mean ±SD median (min-max)	7.52±2.8 8 (1–12)	7.53±3.0 8 (1–12)	8.27±2.8 9 (1–12)	8.90±2.6 9 (1–12)	<0.001
age	mean ±SD median (min-max)	78.2±8.0 78 (64–99)	77.2±7.8 76 (65–101)	74.7±7.1 74 (65–96)	72.3±6.1 71 (65–91)	<0.001
**b. Follow up 1**		teeth class				
		0 1063	1–7 394	8–19 467	20+ 272	p
Physical activity (%)	intense sedentary	17.7 35.6	24.0 26.5	27.3 21.3	29.5 12.9	<0.001
Disability in ADL (%)	0–1 >1 6	61.9 38.1 10.8	29.8 70.2 8.7	74.0 26.0 6.5	84.4 15.6 2.6	<0.001
Mobility-related disability (%)	MRD alone MRD +ADL No MRD	31.2 68.8 0	41.6 58.4 0	49.9 50.1 0	58.5 41.5 0	<0.001
Normal speed march 3m (sec)	mean ±SD median (min-max)	6.54±3.7 5.6 (2.7–39.5)	6.36±3.8 5.4 (2.3–43.0)	6.0±3.3 5.1 (2.7–42.6)	5.7±3.2 4.8 (2.7–30.0)	0.002
Rapid march 3m (sec)	mean ±SD median (min-max)	4.76±3.5 3.9 (1.8–61.6)	4.57±2.7 3.9 (1.5–27.3)	4.35±2.9 3.6 (1.0–39.2)	4.21±3.6 3.5 (1.9–45.0)	0.053
Chair stands 5 times (sec)	mean ±SD median (min-max)	15.1±5.2 13.9 (6.1–40.3)	13.7±4.4 12.8 (4.5–37.4)	13.8±5.7 12.50 (6.9–48.1)	13.8±4.3 12.8 (7.2–33.5)	<0.001
Standing balance test*	mean ±SD % scoring 4	2.87±1.1 40.9	3.04±1.2 50.1	3.19±1.1 57.3	3.47±0.8 65.7	<0.001
Summary SPPB score**	mean ±SD median (min-max)	6.23±3.5 7 (1–12)	6.95±3.6 8 (1–12)	7.47±3.5 8 (1–12)	8.27±2.9 9 (1–12)	<0.001
age	mean ±SD median (min-max)	81.0±7.4 80 (68–102)	79.4±6.9 78 (70–99)	77.5±6.1 76 (69–97)	75.7±5.3 74 (69–93)	<0.001
**c. Follow up 2**		teeth class				
		0 790	1–7 312	8–19 341	20+ 197	p
Physical activity (%)	intense sedentary	11.5 39.7	13.8 31.1	17.63 23.2	21.8 15.2	<0.001
Disability in ADL (%)	0–1 >1 6	58.6 41.4 14.3	66.3 33.7 9.3	70.7 29.3 7.3	83.2 16.8 1.5	<0.001
Mobility-related disability	MRD alone MRD +ADL No MRD	27.4 72.6 0	37.5 62.5 0	41.9 58.1 0	53.3 46.7 0	
Normal speed march 3m (sec)	mean (SD) median (min-max)	6.73±4.7 5.4 (2.5–41.0)	6.40±7.9 5.0 (2.4–105.7)	5.74±3.4 4.9 (2.7–31.0)	5.35±2.1 4.8 (2.5–15.7)	0.002
Rapid march 3m (sec)	mean ±SD median (min-max)	4.84±3.5 4.0 (0.7–46.3)	4.33±2.7 3.7 (1.7–31.0)	4.19±2.5 3.6 (1.9–22.0)	4.08±2.0 3.7 (1.8–16.9)	<0.001
Chair stands 5 times (sec)	mean ±SD median (min-max)	15.6±57 14.3 (6.1–58.1)	14.3±4.7 13.3 (7.0–33.0)	13.7±4.0 12.8 (7.0–31.0)	13.5±3.5 13.0 (7.0–25.4)	<0.001
Standing balance test* score (0–4)	mean ±SD % scoring 4	2.79±1.2 41.9	2.95±1.3 51.0	3.10±1.2 55.4	3.46±0.9 67.5	<0.001
Summary SPPB score**	mean ±SD median (min-max)	5.77±3.6 6 (1–12)	6.62±3.8 8 (1–12)	6.97±3.7 8 (1–12)	8.05±3.1 9 (1–12)	<0.001
age	mean ±SD median (min-max)	81.6±6.7 81 (71–102)	80.2±6.5 80 (71–100)	78.9±5.6 78 (71–96)	77.3±4.9 76 (71–95)	<0.001

*standing balance tests include: Side-by-side, semitandem and tandem, 10 seconds each, with hierarchical difficulty.

** SPPB (short physical performance battery) was calculated by adding the scores of normal speed, rapid march and chair stands (each scored 1–4).

**Table 4:** Results from logistic regression analysis. Separate models with different physical and disability outcome as dichotomized dependent variable were performed with tooth class (0, 1–7, 8–19, and 20+ teeth, reference group was 0 teeth) as exposure variable. ORs and 95% Cis for tooth class 20+ teeth are shown in the table. Models from 1 to 7 are progressively adjusted as described below.

Outcome*	Model 1	Model 2	Model 3	Model 4	Model 5	Model 6	Model 7
SPPB score <9 FU1	**0.60** **0.45–0.79**	**0.68** **0.51–0.91**	**0.60** **0.44–0.82**	**0.60** **0.43–0.84**	**0.56** **0.40–0.79**	**0.60** **0.42–0.86**	0.75 0.52–1.10
SPPB score <9 FU2	**0.55** **0.40–0.75**	**0.64** **0.46–0.89**	**0.58** **0.41–0.83**	**0.54** **0.37–0.78**	**0.50** **0.324–0.74**	**0.55** **0.37–0.81**	**0.59** **0.38–0.91**
Mobility-related disability FU1	**0.51** **0.39–0.67**	**0.58** **0.44–0.77**	**0.63** **0.46–0.85**	**0.62** **0.44–0.87**	**0.57** **0.431–0.81**	**0.62** **0.44–0.88**	**0.67** **0.47–0.97**
Mobility-related disability FU2	**0.50** **0.37–0.67**	**0.58** **0.43–0.80**	**0.58** **0.41–0.82**	**0.63** **0.43–0.91**	**0.58** **0.40–0.85**	**0.65** **0.44–0.95**	0.75 0.49–1.16
Sedentarism FU1	**0.59** **0.42–0.83**	**0.66** **0.47–0.94**	0.70 0.48–1.02	0.68 0.44–1.05	**0.61** **0.39–0.95**	0.65 0.41–1.03	0.68 0.43–1.07
Sedentarism FU2	**0.55** **0.38–0.79**	**0.63** **0.43–0.95**	**0.63** **0.41–0.95**	**0.61** **0.39–0.97**	**0.54** **0.33–0.86**	**0.58** **0.36–0.94**	0.62 0.37–1.03
Disability FU1	**0.62** **0.45–0.86**	**0.68** **0.49–0.96**	0.70 0.49–1.01	**0.57** **0.37–0.87**	**0.53** **0.34–0.81**	**0.54** **0.35–0.84**	**0.54** **0.34–0.85**
Disability FU2	**0.57** **0.40–0.80**	**0.65** **0.46–0.92**	**0.64** **0.44–0.94**	**0.57** **0.37–0.87**	**0.52** **0.34–0.80**	**0.57** **0.36–0.87**	0.72 0.44–1.18
inability to Heavy duty FU1	**0.59** **0.45–0.77**	**0.65** **0.50–0.86**	**0.64** **0.48–0.86**	**0.61** **0.44–0.85**	**0.57** **0.41–0.79**	**0.60** **0.43–0.84**	**0.62** **0.44–0.87**

significant associations are in bold.

* Short physical performance battery (SPPB): 0 = score ≥9; 1 = score <9.

Mobility related disability (MRD): 0 = no; 1 = MRD only; 2 = MRD+ disability in 1 ADL. Class 0 was not present at follow ups.

Sedentarism: 0 = intense-moderate activity; 1 = None.

Disability: 0 Disability in 0–1 ADL; 1 = disability in >1 ADL.

Inability to perform heavy duties: 0 = no; 1 = yes.

Model 1: Sex, age, and BMI-adjusted.

Model 2: Model 1 + comorbidity (>1 of: Myocardial infarction, angina, stroke, congestive heart failure, peripheral arteriopathy, hypertension, chronic obstructive bronchopneumopathy, diabetes, neoplasm), osteoarthritis, osteoporosis, femoral fracture, history of falling, visual impairment, hearing impairment.

Model 3: Model 2 + cognitive impairment, depression, smoking status, alcohol intake, and social variables.

Model 4: Model 3 + serum albumin level, serum vitamin D level, neurological impairment, hand and leg strength.

Model 5: Model 4 + basal physical activity.

Model 6: Model 5 + difficulty at chewing and at swallowing.

Model 7: Model 6 + outcome variable at baseline (for FU1) or F1 (for FU2).

## Discussion

In the present study we explored the association of dental status with physical performance in an elderly population evaluated at baseline and two FUs. Our analyses showed that subjects retaining 20 or more teeth were significantly protected against poor physical performance even after controlling for a number of confounders, including chronic diseases, muscle strength, smoking status, alcohol habits, socioeconomic status, leisure time activity, and difficulty at chewing and at swallowing. These findings are consistent with and support the results of other studies demonstrating an increased risk of disability, institutionalization, and mortality in subjects with a poor dental status [23–25]. While most literature reports are based on self-reported information and self-administered questionnaires, inferences from insurance records, and scatter validated tests [9,26–28], the disease status of our participants was assessed by physicians on the basis of a comprehensive clinical and instrumental examination. Moreover, the Pro.V.A. employed a battery of standardized tests of physical performance that have been shown to be strongly associated with multiple measures of health status in geriatric populations [15–17]. The protective effect of a dentition comprising 20+ teeth was found throughout models that were progressively adjusted. For outcomes linked to mobility, such as SPPB and MRD, the 8–19 teeth class resulted also protective, indicating that even a lower number of teeth can play a beneficial role, as recently described by Matsuyama among English older adults [29]. One would expect a parallel evidence of a detrimental effect of edentulism. Indeed, this condition was significantly associated with low SPPB score at both FUs, but the results were not as consistent as they were for good dentition. We believe that one possible explanation may rely on the heterogenicity of edentulous individuals in which other conditions such as comorbidity play a crucial role that is stronger than dentition. Edentulism is not only the endpoint of multifactorial oral diseases and comorbidities [30] but also the result of physical and cognitive impairments that challenge both personal oral hygiene and professional dental care [31]. Conversely, subjects retaining 20+ teeth represent a “pure” population of healthy elderly in which the number of teeth could be considered as a distinctive trait of physical prowess.

In the early nineties, the 8020 campaign was promoted in Japan to encourage individuals to strive to retain at least 20 natural teeth by the time they reached 80 yrs of age so they could continue to enjoy eating and socializing [32,33]. The idea for the campaign was based on a study showing that people with at least 20 teeth can eat almost any kind of food. The number of pairs of occluding teeth is significantly associated with an increased sense of chewing effectiveness [34] while a low number of masticatory units is associated with an increased risk of CV mortality [35]. In addition, incomplete chewing or rapid swallowing may contribute to esophageal cancer risk [36]. In more recent years, it has been postulated that a shortened dental arch consisting of at least 20 teeth guarantees functionality and durable occlusal stability which, in turn, confer better gait and balance [37,38]. A virtuous circle of events seems to be set off by good dental status that, besides improving nutritional and social aspects, could also ameliorate physical performance which in turn could reduce periodontitis [39] and ameliorate general health.

To our knowledge, this is the first study in which a battery of validated tests was employed to investigate the association between dental status and physical activity in a large population of community-dwelling white elderly of both sexes also characterized for a great number of clinical, laboratory, instrumental, and lifestyle parameters that allow for a wide-ranging adjustment of the relationships. Moreover, the longitudinal design provides insights concerning the persistence of the associations and the predictive role of dental status.

The present work has, however, some limitations. The Pro.V.A. Study’s primary endpoint was the prevalence of osteoarticular and cardiovascular disease in the elderly; dentition and in particular periodontitis were not specifically considered. The study nevertheless engaged clinicians State-board qualified to practice dentistry to collect reliable health-related data of a geriatric population including dental health measures such as the number of remaining teeth and the use of dentures. Interestingly, Desvarieux et al [40] demonstrated that tooth loss is an independent risk factor for carotid atherosclerosis, and they hypothesized it is a more reliable marker of long-term cumulative periodontal disease than measurements of attachment loss. Other studies have also utilized this variable as it can be determined in a straightforward manner and is less prone to measurement errors [41]. Information concerning self-reported difficulties at chewing and/or swallowing was also obtained. The major strength of this work comes from the large amount of variables collected in the Pro.V.A. that allowed us to adjust for a wide number of covariates, namely comorbidity (CVD, osteoarticular, and neurological including depression), muscle strength (assessed for upper and lower limbs), lifestyle (smoking status, alcohol, leisure time activities), and socioeconomical status (education, income, marital status, loneliness). Even if residual confounders (by non-measured factors) cannot be ruled out, we believe that the associations we found are robust because they persisted throughout such an extensive adjustment process.

Among the mechanisms linking tooth loss to disability, nutrition has been demonstrated to play a crucial role, for its influence on muscle strength and central nervous system and -more in general- to health and susceptibility to diseases. The aim of the present work was not to contribute to this important field. However, we addressed the possible role of nutrition introducing in the regression models both nutritional indexes (BMI and serum albumin) and the difficulty at chewing and at swallowing. Nutrition is central in the decline of physical functioning, yet we demonstrated that dentition has an independent role in this transition.

Oral status could be surely considered a marker of fragility as well as fitness in the elderly. Checking dentition during a regular geriatric assessment is not a timeconsuming endeavor and provides extremely useful information about the patient’s general health. It can be considered a cost effective measure given the socioeconomical burden of a rapidly increasing, potentially frail old population worldwide and the importance of health-related preventative care [31]. Similarly, balance tests could effortlessly be implemented in a dental clinic, as they are easy to administer in any outpatient setting and do not require special expertise, instruments and/or locations. Complete or partial edentulism has also been described as a clinical marker of social disadvantage: Peres, in fact, reported that oral conditions disproportionally affect impoverished and socially disadvantaged members of the society [42,43]. It can, in addition, be considered a summative measure of stress (social, emotional, economic, medical, psychological, educational) [34]. Nevertheless, it is still under-recognized as a physical disability although it works together with other more acknowledged impairing conditions in impacting quality of life and longevity. and Public Health campaigns should encourage measures to maintain and reinforce good oral health practices and dental rehabilitation.

Last but not least, we should consider that the aesthetic perfection of the body is widely and energetically pursued in our age and time. Attractive, good-looking teeth are part of a wider search for physical beauty and harmony that can improve not only an individual’s quality of life but also his/her self-esteem [44]. Beyond its aesthetic implications, dental status seems to be able to set off either a vicious or a virtuous circle of events. Poor oral health can lead to malnutrition, deterioration in quality of life, loss of self-sufficiency, and disability while good oral conditions improve nutritional status and social engagement ameliorating general health and quality of life. Within this context, a system foreseeing multiperspective evaluations of health-related variables of senior citizens including dental status could reap many long range rewards for both the individual and the society.
